# First-Trimester Screening Using a Fetomaternal Calculator Versus Maternal History-Based Screening for the Prevention of Preeclampsia

**DOI:** 10.7759/cureus.112490

**Published:** 2026-07-11

**Authors:** Neeru Malik, Sandhya Jain, Dakshika Lochan, Arti Gautum, Rajiv Ranjan, Nikita Madan, Neelu Madan, Sonal Prasad, Parul Talwar, Rishabh Kochar

**Affiliations:** 1 Department of Obstetrics and Gynaecology, Dr. Baba Saheb Ambedkar Medical College and Hospital, New Delhi, IND; 2 Department of Radiology, Dr. Baba Saheb Ambedkar Medical College and Hospital, New Delhi, IND; 3 Obstetrics and Gynecology, Employee's State Insurance Postgraduate Institute of Medical Sciences and Research (ESI PGIMER), Delhi, IND; 4 Obstetrics and Gynaecology, WUS Health Centre, Delhi, IND; 5 Obstetrics and Gynaecology, Bhagwan Mahavir Hospital, Delhi, IND; 6 Pulmonary and Critical Care Medicine, All India Institute of Medical Sciences, Jodhpur, Jodhpur, IND

**Keywords:** aspirin prophylaxis, fetal medicine foundation (fmf) calculator, first-trimester screening, high-risk obstetrics, preeclampsia

## Abstract

Background: Preeclampsia (PE) is a major contributor to maternal and perinatal morbidity in India. While traditional history-based screening is standard, it often lacks sensitivity. This study evaluates the effectiveness of the Fetal Medicine Foundation (FMF) calculator-based screening, utilizing a multi-parametric approach without biochemical markers, against traditional maternal history-based screening in a North Indian population.

Methods: A prospective observational study was conducted at Dr. Baba Saheb Ambedkar Medical College and Hospital (September 2022 to May 2023). Four hundred women at 11 to 13^+6^ weeks gestation (crown-rump length (CRL) 45-84 mm) were randomized into Group A (FMF calculator-based screening using maternal history, mean arterial pressure (MAP), and uterine artery pulsatility index (UtA-PI)) and Group B (maternal history-based screening). High-risk cases (Group A cutoff: ≥1 in 150) received 150 mg of aspirin daily until 36 weeks.

Results: The incidence of preterm PE was significantly lower in Group A (9, 4.9%), compared to Group B (23, 12.4%) (p = 0.011). The total PE incidence was 14 (7.6%) in Group A and 34 (18.3%) in Group B (p = 0.002). Among women initially classified as low risk, 24 (16.0%) in the history-based group developed PE compared with six (4.3%) in the calculator group (p = 0.001). Group A also showed a significant reduction in NICU admissions, i.e., 14 (7.6%) versus 28 (15.1%) in Group B (p = 0.025).

Conclusion: First-trimester screening using the FMF calculator without biochemical markers is significantly more effective than history-based checklists. This protocol facilitates a viable, resource-based screening strategy for low- and middle-income countries.

## Introduction

Preeclampsia (PE) remains a critical challenge in obstetric medicine, representing one of the "deadly triad" of maternal mortality alongside hemorrhage and infection. In India, the clinical burden is substantial, with a reported incidence of 8-10% among pregnant women [1,2]. The disorder is fundamentally characterized by defective trophoblastic invasion of the spiral arteries during the first trimester, leading to failed vascular remodeling, placental insufficiency, and systemic endothelial dysfunction.

Early identification of at-risk patients is essential for preventive intervention. Traditional screening protocols proposed by the American College of Obstetricians and Gynecologists (ACOG) and the National Institute for Health and Care Excellence (NICE) rely on history-based checklists. However, these methods have demonstrated suboptimal performance, often achieving detection rates as low as 39% for preterm PE [3]. By contrast, the Fetal Medicine Foundation (FMF) utilizes a sophisticated competing risks model (as pioneered by Wright et al.). This Bayes' theorem-based algorithm treats PE as a condition that will eventually occur in all women if the pregnancy were to continue indefinitely, estimating the specific risk of delivery with PE before a certain gestational age by integrating biophysical markers [4]. It uses a combination of the prior risk derived from maternal demographic profile and clinical history, mean arterial pressure, and biochemical placental markers (pregnancy-associated plasma protein-A (PAPP-A) and placental growth factor (PlGF)), and mean uterine artery pulsatility index (UtA-PI) to estimate the posterior risk for PE [5].

The prophylactic administration of 150 mg of aspirin, initiated before 16 weeks of gestation, was shown by the Aspirin for Evidence-Based Preeclampsia Prevention (ASPRE trial) to reduce preterm PE by 62% [6]. Aspirin exerts this effect by inhibiting endothelial cyclooxygenase, thereby correcting the prostacyclin-thromboxane A2 imbalance [7]. Given the financial and logistical barriers to biochemical testing (PlGF/PAPP-A) in India, this study validates the FIGO-recommended "resource-based" approach, utilizing only maternal history and biophysical markers (MAP and UtA-PI) to improve perinatal outcomes in a North Indian demographic [8].

## Materials and methods

Study design and clinical setting

This prospective observational study was conducted in the Department of Obstetrics and Gynecology at Dr. B.S.A. Medical College and Hospital, New Delhi, from September 2022 to May 2023.

Participant selection

Inclusion criteria were singleton or twin pregnancies, gestational age 11 to 13^+6^ weeks, and crown-rump length (CRL) between 45 mm and 84 mm. Exclusion criteria were aspirin therapy initiated before 11 weeks; fetal congenital anomalies or intrauterine death at initial scan; history of active peptic ulcer, bleeding disorders, or chronic kidney disease; and history of threatened abortion or vaginal bleeding. Four hundred women were assigned to two groups (n = 200 each) using block randomization with a sealed envelope system.

Group A (FMF calculator-based)

Mean arterial pressure (MAP) was measured in the seated position using a calibrated aneroid sphygmomanometer, and the average of two readings obtained from both arms was recorded. The uterine artery pulsatility index (UtA-PI) was assessed by transabdominal ultrasound at the ascending or descending branch of the uterine artery nearest to the internal cervical os, with an insonation angle of less than 30°. Subsequently, the collected maternal and ultrasound data were entered into the online FMF calculator for preeclampsia risk assessment. A risk threshold of 1 in 150 was used to classify participants as high risk.

Group B (maternal history-based)

Screening followed the standard American College of Obstetricians and Gynecologists (ACOG)/U.S. Preventive Services Task Force (USPSTF) checklists, identifying risk factors, such as nulliparity, obesity (BMI ≥30 kg/m²), family history of PE, previous history of PE, or chronic hypertension.

Intervention

**Figure 1:**
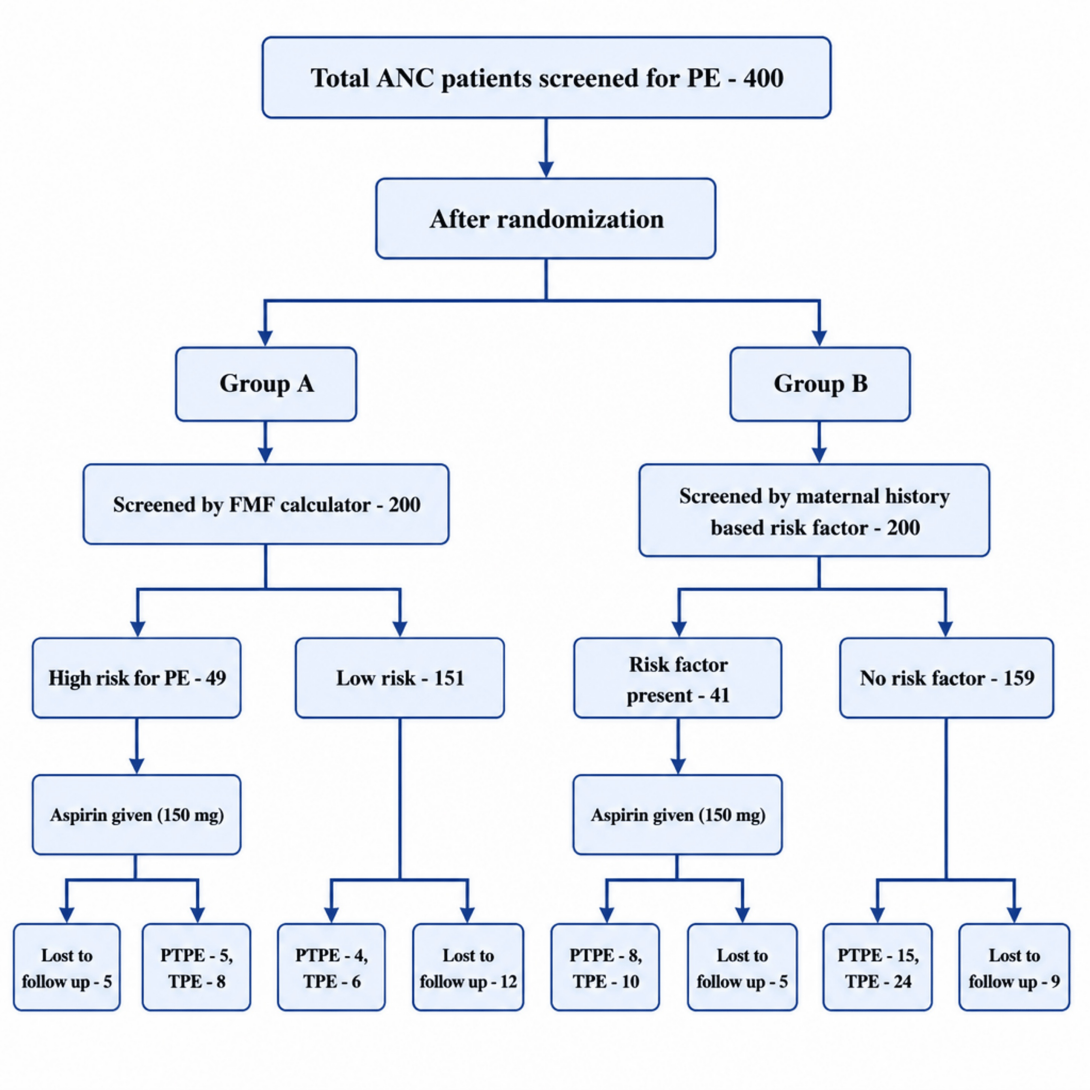
Study design PTPE: preterm preeclampsia, TPE: total preeclampsia, PE: preeclampsia

High-risk participants in both groups received 150 mg of aspirin daily at bedtime [6]. Therapy was initiated before 16 weeks of gestation and continued until 36 weeks. Participants were followed up at 22, 28, and 36 weeks of gestation and through delivery. Primary outcome measures were the development of preterm PE (<37 weeks) and total PE. Secondary outcome measures included fetal growth restriction (FGR), preterm birth, stillbirth, NICU admissions, and adverse effects of aspirin (Figure 1).

The development of preterm preeclampsia is defined in this study as the clinical onset of the disease (new-onset hypertension and/or proteinuria) before 37 weeks of gestation. We acknowledge that the term "preterm PE" is used ambiguously in the existing literature, often used interchangeably to refer to either "preterm-onset PE" (clinical diagnosis before 37 weeks) or "preterm-delivery PE" (delivery before 37 weeks) [9]. We utilised the definition of preterm-onset PE, as the timing of delivery is often subject to institutional induction policies and management strategies. By contrast, the timing of the clinical diagnosis of PE onset more accurately reflects the natural history of the disease. Consequently, the number of clinical cases of preterm-onset PE may differ from the number of preterm births in our cohort, as some patients with early-onset disease may have had deliveries managed at or after 37 weeks.

Statistical analysis

Data were analysed using IBM SPSS Statistics for Windows, version 21.0 (released 2012, IBM Corp., Armonk, NY). Quantitative variables were first assessed for normality using the Shapiro-Wilk test and subsequently compared via unpaired t-tests. Qualitative variables were analysed using chi-square tests; Fisher’s exact test was applied when any expected cell count was less than 5. Predictive accuracy was determined using receiver operating characteristic (ROC) analysis. A ρ-value <0.05 was considered statistically significant.

## Results

Of the 400 women initially randomized (200 in each group), 31 were lost to follow-up or excluded from the final analysis, including 17 participants in Group A and 14 participants in Group B. Consequently, outcome data were available for 183 women in Group A and 186 women in Group B.

Demographic profile

There were no statistically significant differences in age, weight, or BMI between Group A and Group B, ensuring baseline comparability between the two cohorts (Table 1).

**Table 1:** Comparison of demographics in Groups A and B BMI: body mass index. * Unpaired t-test.

Parameter	Group A (Mean ± SD)	Group B (Mean ± SD)	p-value*	t-value
Age (years)	25.26 ± 4.13	25.72 ± 4.27	0.288	-1.602
Weight (kg)	49.46 ± 5.23	50.41 ± 5.45	0.079	-1.761
BMI (kg/m²)	20.15 ± 3.37	20.67 ± 3.3	0.123	-1.543

Incidence of preeclampsia

The FMF calculator-based approach resulted in a significant reduction in PE rates compared to the traditional history-based checklist (Table 2).

**Table 2:** Comparison of the incidence of preeclampsia in Groups A and B PE: preeclampsia. + Chi-square test.

Outcome	Group A (n = 183)	Group B (n = 186)	p-value^+^	χ²
Preterm PE	9 (4.9%)	23 (12.4%)	0.011	6.44
Total PE	14 (7.6%)	34 (18.3%)	0.0024	9.18

Subgroup analysis: the "low risk" gap

A critical finding emerged among women initially classified as "low risk." In Group B, 24 (16.0%) women initially classified as low risk subsequently developed preeclampsia, compared with six (4.3%) women in Group A (p < 0.001), highlighting the inadequacy of history-based screening alone.

Fetal and neonatal outcomes

NICU admissions were significantly lower in the calculator-screened group (Table 3).

**Table 3:** Comparison of fetal and neonatal outcomes in Groups A and B NICU: neonatal intensive care unit. + Chi-square test. # Significant at p = 0.025; remained significant at p = 0.037 after Yates correction. &: Fisher's exact test.

Neonatal outcome	Group A (n = 183)	Group B (n = 186)	p-value^+^	χ²
Preterm birth	14 (7.6%)	15 (8.1%)	0.882	0.02
Stillbirth	0 (0.0%)	2 (1.1%)	0.498^&^	Not applicable
NICU admission	14 (7.6%)	28 (15.1%)	0.025^#^	5.02
Fetal growth restriction (FGR)	10 (5.4%)	12 (6.4%)	0.688	0.16

Safety and predictive accuracy

Aspirin Safety

No adverse effects, such as peptic ulcers or placental abruption, were observed in either group.

ROC (Receiver Operating Characteristic) Analysis (Group A)

**Figure 2:**
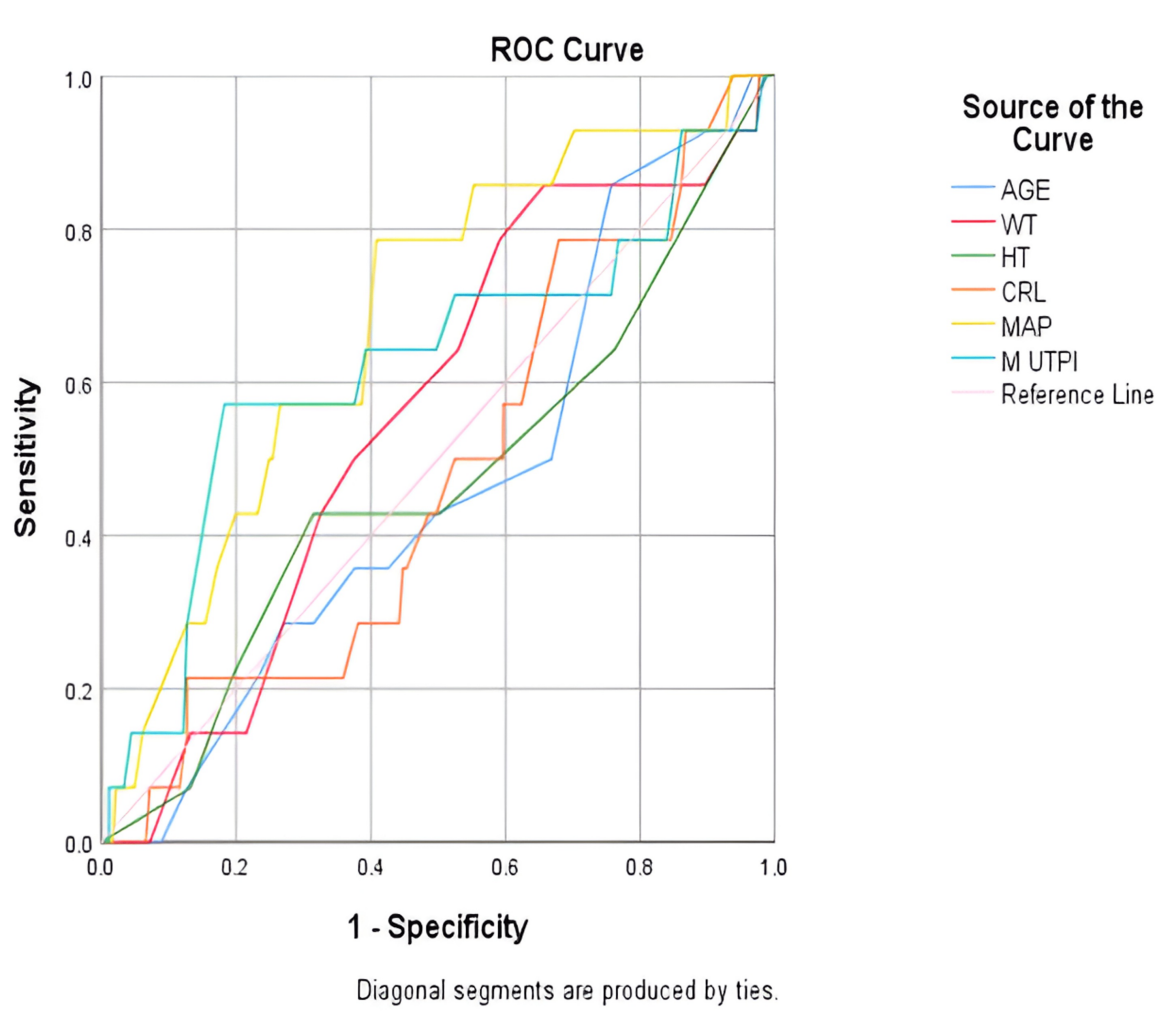
ROC analysis for the predictors of total preeclampsia in Group A WT: weight, HT: height, CRL: crown-rump length, MAP: mean arterial pressure, MUTPI: mean uterine PI

**Figure 3:**
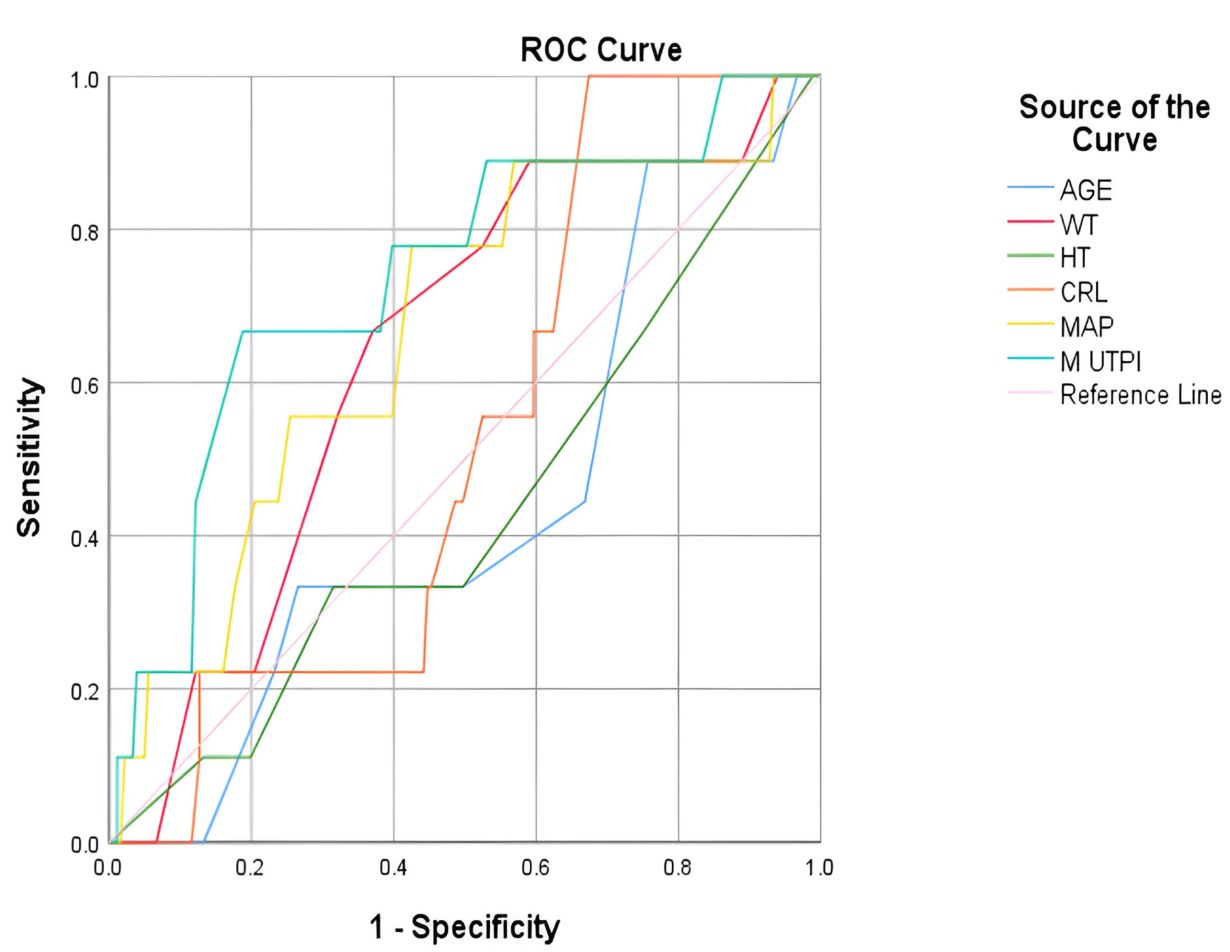
ROC analysis for the predictors of preterm PE in Group A PE: preeclampsia, WT: weight, HT: height, CRL: crown-rump length, MAP: mean arterial pressure, MUTPI: mean uterine PI

UtA-PI was a significant predictor for preterm PE (AUC 0.739, p = 0.016), while MAP was significant for total PE (AUC 0.681, p = 0.024) (Figures 2, 3).

ROC Analysis (Group B)

**Figure 4:**
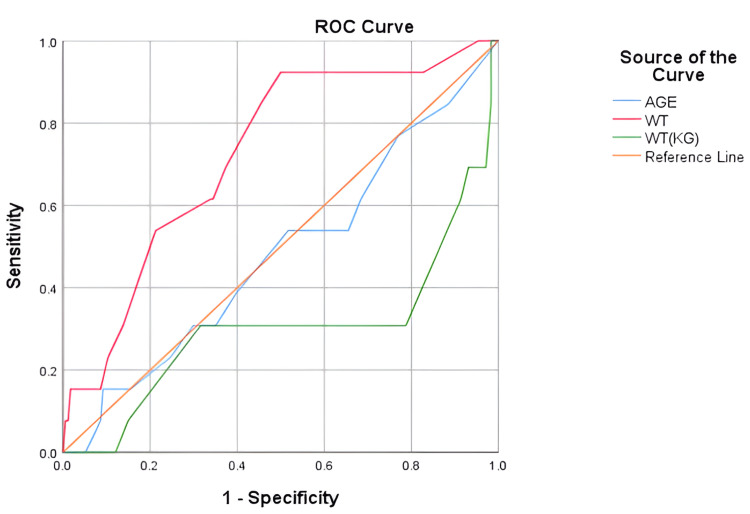
ROC analysis for the predictors of preterm PE in Group B ROC: receiver operating characteristic, PE: preeclampsia

Maternal weight was the only significant predictor for preterm PE (AUC 0.724, p = 0.007) (Figure 4).

## Discussion

The current study demonstrates several methodological and clinical advantages over existing literature evaluating first-trimester preeclampsia screening. A primary strength of our research is the use of a randomised, parallel comparative design that directly contrasts an integrated fetomaternal calculator with standard maternal history-based screening. By contrast, large-scale regional studies, like the Samrakshan program [10], lacked a comparative parallel group, relying instead on previous year's national statistics from the National Family Health Survey-4 (NFHS-4 data) to calculate reductions in preeclampsia rates. By observing two randomised groups concurrently, our study provides a direct quantification of the calculator's superiority over the standard OPD practices used in busy government hospitals.

Our prospective randomisation also eliminated baseline disparities between groups that have confounded previous research. For example, Rezende et al. (2017) grouped women retrospectively based on hospital records and found significant baseline differences in maternal age, weight, and history of preeclampsia [11]. Our concurrent randomisation ensured no statistically significant differences in these variables between the screened groups. In addition, while Rezende et al. excluded complex cases, like systemic lupus erythematosus (SLE) and antiphospholipid syndrome (APS) [11], our study actively included these cases, making our findings more representative of real-world clinical populations.

Furthermore, our research provides more robust evidence of screening efficacy compared to other recent international trials. Lourenço et al. (2020) [12] compared their screening results against historical hospital data collected before the intervention, while Prasad et al. (2021) [13] did not compare their preeclampsia reduction rates with a control group or previous data at all. By directly comparing our intervention group against a concurrently observed control group, we conclusively demonstrated a statistically significant reduction in both preterm preeclampsia (4.9% vs. 12.4%) and total preeclampsia (7.6% vs. 18.3%).

Another critical advantage of our study is the pragmatic adaptation of risk cut-offs to suit local healthcare realities. While Lourenço et al. utilised a one-in-50 cut-off [11] and both Prasad et al. [13] and Johnson et al. [14] used a one-in-100 threshold, we deliberately selected a broader one-in-150 cut-off for recommending aspirin prophylaxis. This tailored approach is superior for the Indian context, as it specifically accounts for the higher background prevalence of preeclampsia, lower uptake of antenatal services, and significant patient dropout rates in the health system.

Like the Samrakshan study [10], we actively chose to exclude biochemical markers due to their lack of affordability and accessibility at a large scale. However, we advanced this approach by validating that a free, online fetomaternal calculator, combined with biophysical markers, specifically the mean uterine artery pulsatility index (AUC 0.739), is sufficient to accurately predict preterm preeclampsia.

Finally, our study expands on perinatal outcomes that were overlooked in similar research. While the Samrakshan study [10] did not report on NICU admissions, our trial demonstrated that fetomaternal calculator screening leads to a statistically significant reduction in NICU admissions (7.6% vs. 15.1%). Combined with the calculator's superior ability to identify true low-risk pregnancies, reducing the incidence of preeclampsia among women classified as low risk from 16% to 4.3%, our study establishes a highly effective, resource-conscious protocol that definitively surpasses traditional checklist-based methods.

## Conclusions

The implementation of the first-trimester FMF calculator-integrating maternal history, MAP, and UtA-PI-is associated with a significant reduction in the rates of both preterm and total preeclampsia within our study cohort compared to traditional history-based screening. This multi-parametric approach also demonstrates potential for improving neonatal outcomes by reducing NICU admissions.

Based on these observations, early first-trimester risk stratification between 11 and 13^+6^ weeks appears to offer substantial clinical utility. Standardizing MAP measurements and incorporating UtA-PI assessment during routine first-trimester scans may serve as an effective, resource-conscious screening framework, particularly in resource-limited settings where biochemical markers are unavailable. However, while these results are encouraging, they represent data from a single center. These findings ultimately support the potential value of this screening strategy and justify further validation in larger, multicenter trials to confirm generalizability before widespread clinical implementation is mandated.
